# *Drosophila* as a Model for MECP2 Gain of Function in Neurons

**DOI:** 10.1371/journal.pone.0031835

**Published:** 2012-02-21

**Authors:** Fernando Vonhoff, Alison Williams, Stefanie Ryglewski, Carsten Duch

**Affiliations:** School of Life Sciences, Arizona State University, Tempe, Arizona, United States of America; Columbia University, United States of America

## Abstract

Methyl-CpG-binding protein 2 (*MECP2*) is a multi-functional regulator of gene expression. In humans loss of *MECP2* function causes classic Rett syndrome, but gain of *MECP2* function also causes mental retardation. Although mouse models provide valuable insight into *Mecp2* gain and loss of function, the identification of MECP2 genetic targets and interactors remains time intensive and complicated. This study takes a step toward utilizing *Drosophila* as a model to identify genetic targets and cellular consequences of *MECP2* gain-of function mutations in neurons, the principle cell type affected in patients with Rett-related mental retardation. We show that heterologous expression of human MECP2 in *Drosophila* motoneurons causes distinct defects in dendritic structure and motor behavior, as reported with MECP2 gain of function in humans and mice. Multiple lines of evidence suggest that these defects arise from specific MECP2 function. First, neurons with MECP2-induced dendrite loss show normal membrane currents. Second, dendritic phenotypes require an intact methyl-CpG-binding domain. Third, dendritic defects are amended by reducing the dose of the chromatin remodeling protein, osa, indicating that MECP2 may act via chromatin remodeling in *Drosophila*. MECP2-induced motoneuron dendritic defects cause specific motor behavior defects that are easy to score in genetic screening. In sum, our data show that some aspects of MECP2 function can be studied in the *Drosophila* model, thus expanding the repertoire of genetic reagents that can be used to unravel specific neural functions of MECP2. However, additional genes and signaling pathways identified through such approaches in *Drosophila* will require careful validation in the mouse model.

## Introduction

Methyl-CpG-binding protein 2 (*MECP2*) is a multifunctional transcriptional regulator involved in chromatin remodeling. Loss of MECP2 function mutations cause classic Rett Syndrome (RTT) [1], [2], an X-linked, dominant, progressive, neuro-developmental disorder [3], [4]. Patients with RTT suffer from cognitive, language, motor conditions, and seizures [5], [6]. However, *MECP2* duplication is a frequent case of mental retardation and progressive neurological symptoms in males [7], [8], [9], and overexpression of MECP2 in the developing mouse brain also causes progressive neurological disorder [10], [11].

The MECP2 protein contains at least five distinct functional domains (NTD, ID, MBD, TRD, and CTDα) which either bind DNA autonomously or regulate MBD (methyl-CpG binding) function [12]. Historically, MECP2 is viewed as a transcriptional repressor that localizes to chromatin by binding to CpG dinucleotides to regulate gene expression through interactions with histone deacetylases and other cofactors [13], [14], [15], [16]. However, MECP2 can also activate transcription [17], associates also with un-methylated DNA [12], [18], [19], has chromatin compaction and RNA splicing functions [20], [21], [22], and several MECP2 interacting proteins have been identified [2]. Therefore, multiple MECP2 functions might be mediated by interactions with diverse co-factors and by binding to both methylated and non-methylated DNA, consistent with the wide range of phenotypes observed in patients with RTT.

Although *Mecp2* mouse models recapitulate RTT phenotypes [23], [24], [25], [26], [27] and have provided valuable mechanistic insight into neuronal defects caused by *Mecp2* mis-regulation, such as axon targeting [28], synaptic [29], [30], and dendritic [31] defects, the identification of MECP2 functions and target genes in this system is time intensive and complicated.

Facile genetic tools [32], short generation times, and a high degree of conservation in fundamental cell biological pathways [33] make *Drosophila* a powerful model to study molecular and cellular mechanisms underlying mental retardation [34], [35], [36], [37]. It is not expected that *Drosophila* will recapitulate all details of Rett-related pathophysiology since its genome is sparsely methylated [38] and does not contain an ortholog of human *MECP2*. However, multiple MECP2 interactors and most components of the chromatin machinery have well conserved orthologs in flies [39]. In transgenic flies that express human MECP2, the protein associates with chromatin, modifies the transcription of multiple genes, and is phosphorylated at serine 423, as in mammals [40]. Significantly, reported consequences of a MECP2 gain-of-function in *Drosophila* are developmental defects and motor dysfunctions, suggesting close parallels with RTT phenotypes [40].

Our current study expands genetic and behavioral proof of principle for studying MECP2 gain-of-function phenotypes in *Drosophila* [40] to the level of central neurons. MECP2 expression in identified *Drosophila* motoneurons results in dendritic defects but normal membrane properties. MECP2-caused dendritic defects require an intact MBD domain, can be ameliorated by dose reduction of the chromatin remodeling protein osa, and cause specific motor behavioral defects, thus indicating that the *Drosophila* model is useful to unravel some aspects of MECP2 function in neurons.

## Results

**Figure 1:**
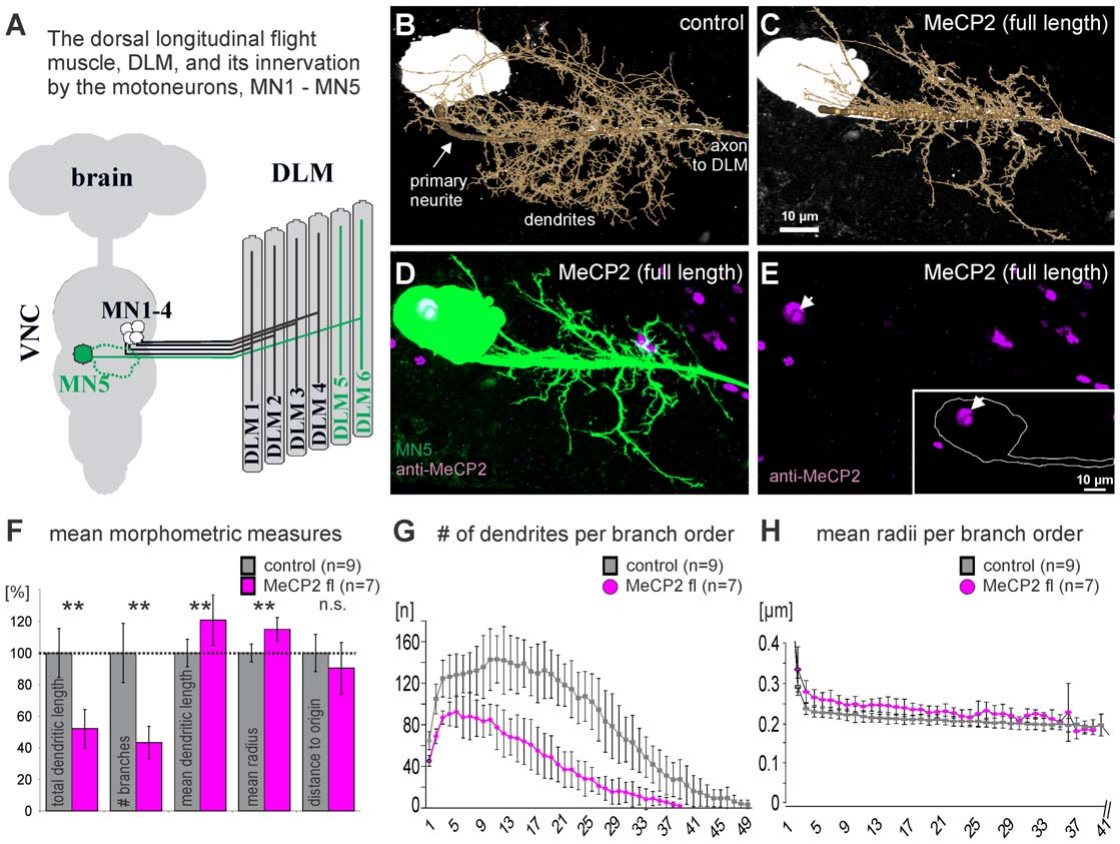
Heterologous expression of MECP2 causes dendritic defects in Drosophila motoneurons. (A) Schematic drawing of location of MN1-5 in the *Drosophila* nervous system and their innervation of the dorsal longitudinal flight muscle (DLM) fibers. MN5 is depicted in green, and MN5 dendritic projection in the dorsal mesothoracic neuromere is demarked by a dotted green line (B) Overview of MN5 structure in a representative control animal. A geometric reconstruction of MN5 dendritic structure is superimposed on the projection view. (C) Overview of MN5 dendritic structure following targeted expression of full-length human MECP2. Geometric reconstruction superimposed on projection image of MN5. (D) Double staining of MN5 (green) and anti-MECP2 immunolabeling (magenta) shows MECP2 localization to MN5 nucleus (white) and some other nuclei of neurons with *C380-GAL4/UAS-MECP2* expression. (E) Same as (D) but anti-MECP2 immunolabeling only to show that no MECP2 protein was detected through MN5 processes. Inset depicts anti-MECP2 immunostaining in a representative single optical section through MN5 soma and primary neurite. MN5 outline is demarked by white line, and white arrow demarks MN5 nucleus. MECP2 protein could not be detected in any part of MN5 except the nucleus. (F) Quantitative metric measures of dendritic structure in MN5 from controls (gray bars) and in MN5 with MECP2 expression (magenta). Values are normalized to mean control values (dotted line). Arrows indicate statistical significant differences (Students T-test, p≤0.01). Error bars indicate standard deviation. (G and H) Mean number of dendritic branches (G) and mean dendritic radius in controls (gray squares) and following MECP2 expression (magenta circles) over branch order. Error bars indicate standard deviation. Axis in (H) is clipped at branch order 41 because only few dendrites of higher branch orders exist (see G).

This study used the individually identified flight motoneuron, MN5, to study effects of targeted expression of human MECP2 variants in *Drosophila* neurons on dendritic structure and membrane properties. As is the unique advantage of all individually identified neurons in invertebrate preparations, MN5 can be unambiguously identified in every individual fly, and it exhibits a characteristic morphology, membrane properties, and a distinct function albeit integrated into a network. MN5 is one of only five MNs innervating the dorsal longitudinal flight muscle (Fig. 1A, DLM) [41], [42] which provides the main force for wing downstroke during *Drosophila* flight. MN5 is a large monopolar neuron with its soma located in the mesothoracic neuromere of the *Drosophila* ventral nerve cord (VNC), on the contralateral side with respect to its target muscle (Fig. 1A), [43]. All MN5 dendrites develop *de novo* during pupal life [42], thus allowing for studies of postembryonic dendritic growth. MN5 dendrites span the dorsal neuropil of the second thoracic neuromere of the *Drosophila* ventral nerve cord (Fig. 1A, dotted green line), and we have shown previously that MN5 dendritic structure shows reasonably low variation among control animals, which allows for quantitative studies of the effects of genetic manipulation [44]. In the adult fly, the dendritic field of MN5 comprises more than 4000 dendritic branches making up for more than 6500 µm total length. In addition, we have analyzed firing responses [43] and membrane currents [45] in control MN5.

We used the UAS-GAL4 system to express three different forms of human MECP2 using previously constructed transgenes (kindly provided by Dr. J Botas, Baylor College of Medicine, Houston, Texas) under the control of motoneuron-specific GAL4 drivers (*C380-GAL4*; see methods). The first is full-length human *MECP2*, and the other two are *MECP2* alleles mutant in the MBD domain. The *R106W* allele is a missense mutation that creates a non-functional methyl-CpG-binding domain (MBD) [46]. In the Δ*166* mutation the MBD and N-terminal portion of the protein are removed.

### Full-length human MECP2 specifically causes dendritic defects but does not impair normal membrane excitability in Drosophila motoneurons

Intracellular fills of MN5 in control animals with subsequent quantitative dendritic architecture reconstruction (Fig. 1B) yielded the same values for MN5 dendritic tree structure as previously published [44], but expression of full-length human *MECP2* in MN5 clearly affected MN5 dendritic structure (Figs. 1C, D). Targeted expression of full-length human *MECP2* in MN5 and few other neurons (see methods for expression patterns of *C380-GAL4; Cha-GAL80*) resulted in localization of MECP2 protein to the nuclei of these neurons, as demonstrated by MECP2 immunocytochemistry (Figs. 1D, E, magenta, see white arrow for MN5 nucleus). Careful inspection of single optical sections through MN5 nucleus and dendrites (see inset in figure 1E) showed that no anti-MECP2 immunopositive label was detectable outside the nucleus.

Quantitative comparison of MN5 dendritic structure in controls (Fig. 1B) and following over-expression of MECP2 (Figs. 1C, D) caused a significant decrease in the number of branches by 60% (from 4000±90 in controls to 1734.85±713) which resulted in significantly decreased total dendritic length by about 50% (Fig. 1F, from 6517±471 µm in controls to 3490±816). By contrast, the mean lengths of the individual dendritic branches was slightly (20%) but significantly increased (Fig. 1F, from 1.69±0.13 µm in controls to 2.04±0.26 µm). Therefore, dendritic branch elongation was not impaired but new branch formation was strongly reduced by MECP2 expression. Average dendritic radii were also significantly increased following MECP2 expression (Fig. 1F, from 0.23±0.01 µm in controls to 0.26±0.01 µm). However, dendritic territory borders were not affected as indicated by normal average distances of the dendrites to their origin (Fig. 1F, 17.7±2.1 µm in controls and 16.5±2.65 µm). Branch order analysis (Figs. 1G, H) revealed that these dendritic phenotypes were not restricted to specific branch orders, indicating that MECP2 affected new dendritic branch formation and growth during all stages of postembryonic dendritic growth. Similar conclusions resulted from Sholl analysis which measures dendritic lengths or dendrite numbers in concentric 3-dimensional spheres at different distances around the origin of the dendritic tree (not shown). MN5 dendritic defects as resulting from gain-of-function of MECP2 were not a result of developmental delay because intracellular staining of MN5 in three, five, or ten days old adult flies did not reveal additional dendritic branches (not shown). By contrast, in progressively older flies MECP2-induced dendritic defects seemed increasingly more severe, although we did not quantify this observation.

**Figure 2:**
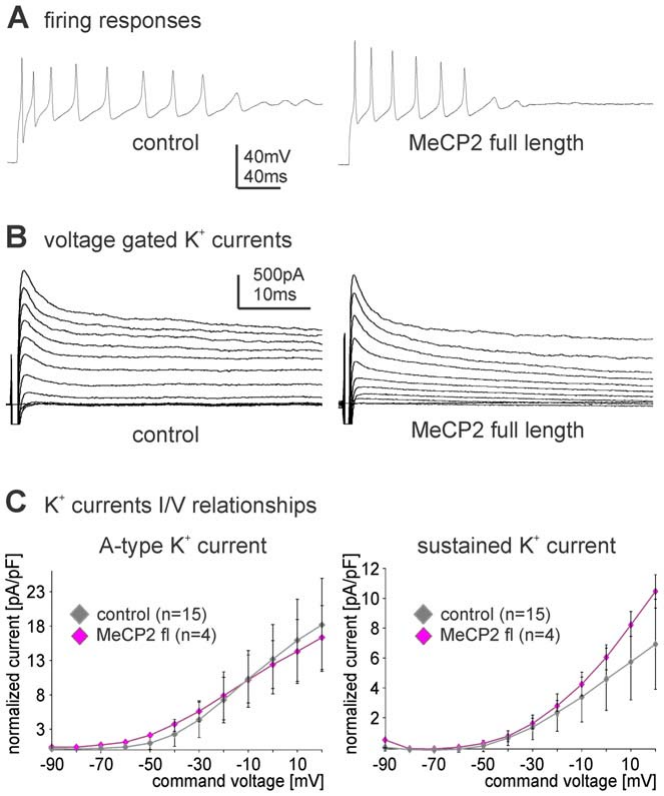
Heterologous expression of MECP2 does not affect electrophysiological properties of Drosophila motoneurons. (A) Comparison of typical MN5 firing responses to 300 pA of somatic current injection in a representative control animal (left trace) and following targeted expression of full-length human *MECP2* under the control of *C380-GAL4* (right trace). (B) Voltage dependent potassium currents in MN5 as induced by command voltage steps from a holding potential of −90 mV to 20 mV in increments of 10 mV and with cadmium and TTX in the bath solution to block sodium and calcium inward currents. Traces of control animals (left) and following targeted expression of MECP2 (right) reveal qualitatively similar transient A-type current and sustained delayed rectifier like potassium outward currents. (C) Current/Voltage relationships for A-type (left) and sustained delayed rectifier (right) potassium currents are quantitatively similar in controls (gray diamonds) and following expression of MECP2 (magenta diamonds). Error bars represent standard deviations.

Electrophysiological recordings in current and in voltage clamp mode showed that targeted expression of human MECP2 in MN5 did not affect firing properties or potassium membrane currents. Current clamp recordings revealed no obvious differences in MN5 firing responses to somatic current injections between controls and following MECP2 expression (Fig. 2A). *In situ* voltage clamp recordings from MN5 under cadmium and TTX revealed no obvious differences in transient A-type or sustained delayed rectifier type voltage activated potassium currents in controls and following MECP2 expression (Figs. 2B, C). Quantification of A-type and delayed rectifier potassium currents revealed no significant differences in I/V–relationships between controls and following MECP2 expression (Fig. 2C). In sum, over-expressed human MECP2 localized to the nucleus in a *Drosophila* motoneuron and significantly impaired new dendrite formation resulting in a reduction of total dendritic length by 50 percent. However, full-length MECP2 did not affect normal development of membrane excitability, did not alter dendritic territory borders, and did not impair dendritic branch elongation. This indicated that over-expression of MECP2 specifically impaired dendritic branching but did not have overall deleterious effects on motoneuron physiology.

### Dendritic defects caused by human MECP2 in Drosophila motoneurons require normal MBD function

**Figure 3:**
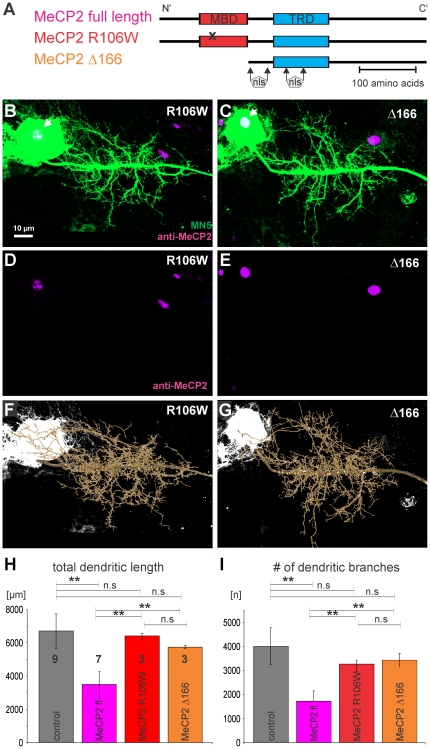
Heterologous expression of MECP2 with MBD defects does not affect Drosophila motoneuron dendrite development. (A) Schematic drawings of full-length human MECP2 (magenta) with intact menthyl-CpG-binding domain (MBD) and intact transcriptional repression domain (TRD). The R106W mutation (red) carries a point mutation (see x) that causes a non-functional MBD. The Δ166 mutation (orange) has a truncated MBD and N-terminus. TRD is intact in all three alleles. Nuclear localization sequences (nls) have been reported in the inter-domain region at residues 174 and 190 and also in the TRD domain between residues 255 and 271, and are intact in all three alleles. (B, D, F) Intracellular labeling of MN5 following R106W expression under the control of *C380-GAL4* (B) and subsequent geometric reconstruction (F) do not reveal obvious dendrite defects in MN5. (D) MECP2 immunolabeling following targeted R106W expression indicates strict nuclear localization (see also white arrow in B). (C, E, G) Intracellular labeling of MN5 following Δ166 expression under the control of *C380-GAL4* (C) and subsequent geometric reconstruction (G) do not reveal obvious dendrite defects in MN5. (E) MECP2 immunolabeling following targeted R106W expression indicates strict nuclear localization (see also white arrow in C). (H) Averages of total dendritic length in controls (gray bars), and following expression of full-length MECP2 (magenta), R106W (red), and Δ166 (orange). (I) Average numbers of dendritic branches in controls (gray bars), and following expression of full-length MECP2 (magenta), R106W (red), and Δ166 (orange). In (H) and (I) error bars indicate standard deviation, asterisks demark statistical significance at p≤0.05 (ANOVA with Newman Keuls posthoc test).

Next, we confirmed that dendritic defects as caused by targeted expression of human *MECP2* in *Drosophila* motoneurons were dependent on known molecular functions for MECP2, and not due to non-specific effects that can potentially result from the expression of a non-endogenous protein. To test whether normal MECP2 protein function was required for the observed effects on dendrite development, we expressed two *MECP2* transgenes with non-functional methyl-CpG-binding domains (MBD; Fig. 3A) in MN5 under the control of the same *C380-GAL4* driver that yielded dendritic defects when used to express full-length *MECP2*. MBD domains are required for the two key mechanisms of chromatin regulation in eukaryotes, C5 methylations of DNA at cytosines and posttranslational histone modifications [47]. Expression of *UAS-MECP2* with either a mis-sense mutation that creates a non-functional MBD (Fig. 3B; *R106W* allele) [46], or with a truncated MBD and N-terminal portion (Fig. 3C; Δ*166* allele) did not cause any obvious dendritic defects (Figs. 3B to G). As for full-length *MECP2* (see above) strict nuclear localization of MECP2 was observed for the *R106W* and the Δ*166* alleles (Figs. 3D, E; see also white arrows in figures 3B, C). Quantification of total dendritic length (Fig. 3H) and the number of dendritic branches (Fig. 3I) demonstrated that no significant differences existed between controls or following targeted expression of MECP2 with defective MBD (Figs. 3H, I; ANOVA with Newman Keuls post hoc testing, p>0.2). By contrast, expression of full-length human MECP2 caused less total dendritic length (Fig. 3H) and significantly fewer branches (Fig. 3I) than expression of either *R106W* or Δ*166* (ANOVA with Newman Keuls post hoc testing, p<0.01). Therefore, dendritic phenotypes induced by targeted expression of human MECP2 in *Drosophila* neurons required an intact MBD, indicating specific action and not unspecific toxic effects of *MECP2* gain-of-function in *Drosophila* motoneurons.

### Dendritic defects in Drosophila motoneurons caused by gain-of-function of human MECP2 can be ameliorated by reducing the dose of the BAF250 homolog, osa

**Figure 4:**
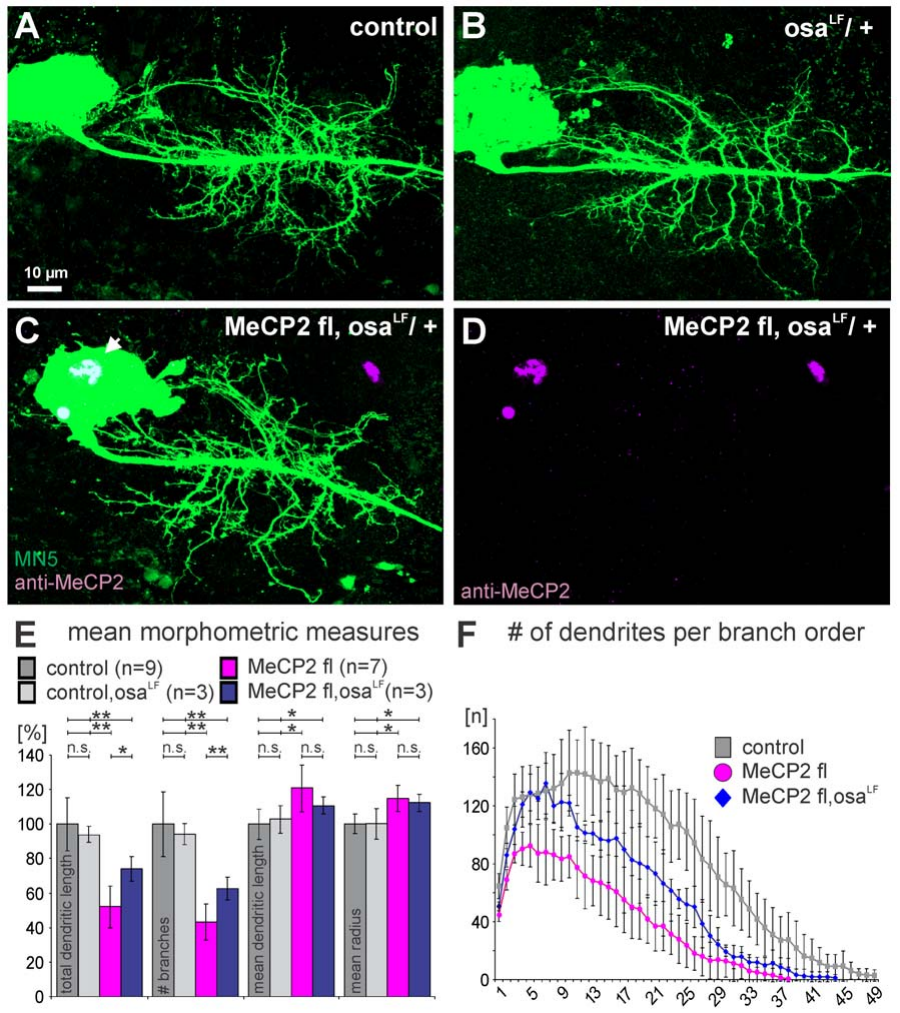
MECP2-caused dendrite defects are partially ameliorated by a reduction in osa dose. (A) Projection view of a representative intracellular staining of MN5 in a control animal. (B) Projection view of a representative intracellular staining of MN5 in an *osa* heterozygous mutant background does not reveal obvious differences in dendritic structure as compared to control. (C) Projection view of a representative intracellular staining of MN5 with heterologous expression of full-length MECP2 in an *osa* heterozygous mutant background does not show similar dendritic defects as compared to MECP2 expression in a wildtype *osa* background (see figures 1C, D). (D) MECP2 immunopositive label (magenta) was restricted to the nucleus (see also white arrow in C). (E) Quantitative metric measures of dendritic structure in MN5 from controls (dark gray bars), MN5 in an *osa* heterozygous mutant background (light gray bars), from MN5 with MECP2 expression (magenta), and from MN5 with MECP2 expression in an *osa* heterozygous mutant background. Values are normalized to mean control values. Arrows indicate statistical significance (ANOVA with Newman Keuls posthoc test, p≤0.01). Error bars indicate standard deviation. (F) Mean number of dendritic branches over branch order in controls (gray squares), following MECP2 expression (magenta circles), and following MECP2 expression in an *osa* heterozygous mutant background (blue). Error bars indicate standard deviation.

Since *Drosophila* DNA is only sparsely methylated, interactions of the MBD of MECP2 with C5 methylations of DNA at cytosines seem unlikely (see discussion). Alternatively, the MECP2 MBD might interact with posttranslational histone modifications [47]. This is in agreement with previous findings that reduction of osa function can amend behavioral defects as induced by pan neuronal expression of human MECP2 in flies [40]. Osa (human homolog is BAF250) is a member of the SWI/SNF complex, a class of trithorax proteins involved in chromatin remodeling [48]. To test whether MECP2-induced dendritic defects require normal function of an intact BAF complex (ATP-dependent chromatin remodeling complex) we expressed full-length human *MECP2* in MN5 in a heterozygous *osa* mutant background, which should lower the dose of functional osa protein. Intracellular labeling of MN5 in a heterozygous *osa* mutant background (Fig. 4B) and subsequent quantification (Fig. 4E) showed that a reduction in osa dose did not alter dendritic structure as compared to controls (Figs. 4A, E). However, the heterozygous *osa* mutant background significantly ameliorated MECP2-induced dendritic effects in MN5 (Figs. 4 C, E, F). The strict nuclear localization of MECP2 was not altered by a reduction in osa function (Fig. 4D). Although total dendritic length and the number of dendrites were significantly lower as compared to controls, MN5 contained significantly more dendrites and a larger total dendritic length if expressed in the presence of reduced osa function as compared to expression of MECP2 in controls (Fig. 4E). Therefore, dendritic defects as caused by *MECP2* gain-of-function can be partially rescued by a reduction in osa function, thus indicating functional interactions of MECP2 and osa. This was also reflected by branch order analysis. Expression of full-length human MECP2 in an *osa* heterozygous mutant background resulted in fewer dendrites through all branch orders higher than eight as compared to controls (Fig. 4F), but it resulted in more dendrites per branch order as compared to expression of full-length MECP2 in a wild type background (Fig. 4F). By contrast, increased mean length and radius of individual dendritic branches as induced by targeted expression of MECP2 were not rescued in an *osa* mutant background. In sum, these genetic interaction experiments show that MECP2-induced MN5 dendritic branch formation defects require normal osa function, indicating that the MB domain of MECP2 may interact with the ATP-dependent chromatin remodeling BAF complex (see discussion).

### Motor behavioral consequences of MECP2-induced dendritic defects

**Figure 5:**
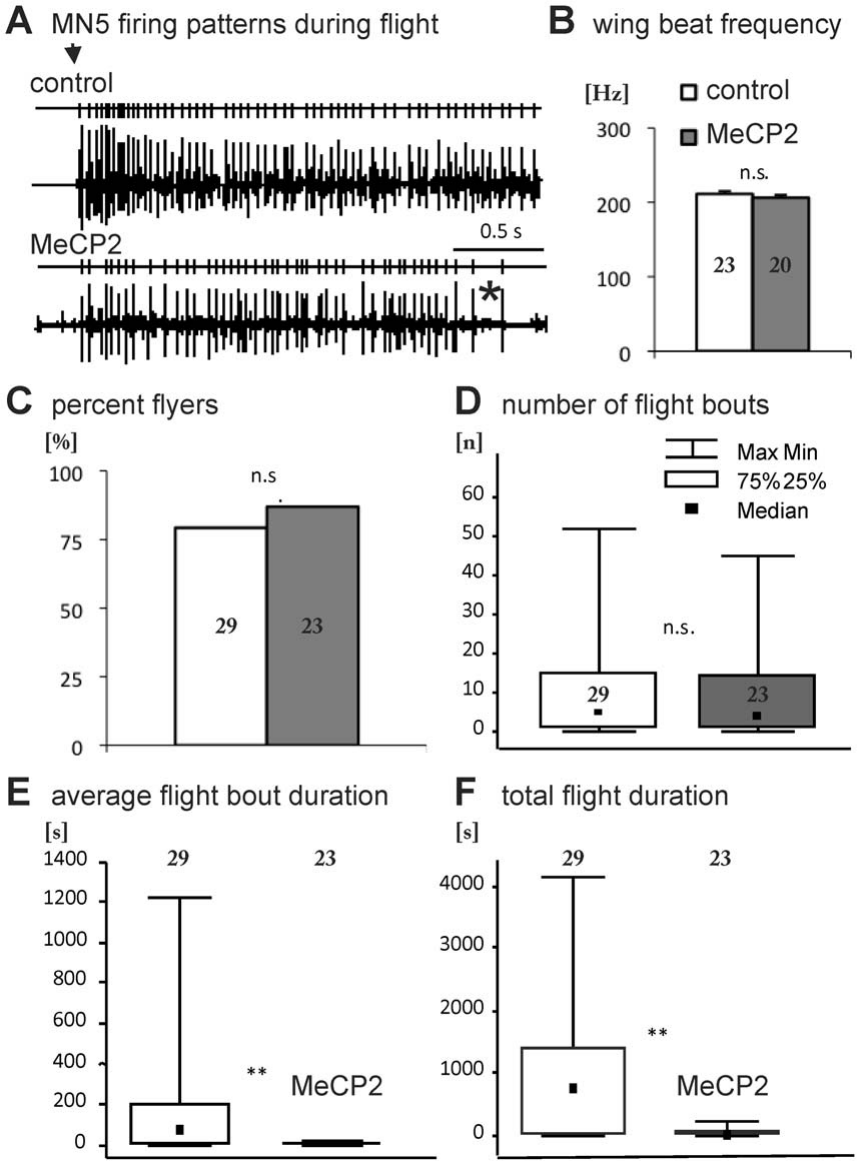
MECP2-induced motoneuron defects result in specific motor behavioral deficiencies. (A) Representative extracellular recording of MN5 firing patterns during flight in a control (upper trace) and in fly expressing MECP2 in a subset of neurons, including MN5 (*C380-GAL4, UAS-mcd8-GFP; Cha-GAL80/UAS-MECP2*; lower trace). Traces above the recordings resemble spike counts. Black arrow demarks start of flight, and black asterisk demarks time point of flight stop in *MECP2* fly. (B) Average in-flight wing beat frequencies of control (white bar) and *MECP2* flies (grey bar). Error bars represent standard error. (C) Percentage of control (white bar) and *MECP2* flies (grey bar) engaging into flight upon a wind stimulus. (D) Numbers of flight bouts performed by control (white bar) and by *MECP2* flies (grey bar) in response to re-occuring wind stimuli (see methods). Data are presented as median and quartiles. Error bars represent minimum and maximum values. (E and F) Total duration of all consecutive flight bouts (E) and average duration of individual flight bouts (F) in control (white bar) and in *MECP2* flies (grey bar). Data are presented as median and quartiles. Error bars represent minimum and maximum values. ** demarks p<0.01, Mann and Whitney U-test.

Human RTT patients suffer from motor deficits, and mouse models recapitulate motor dysfunctions. Similarly, Cukier et al. [40] reported that expression of full-length human MECP2 in all cholinergic neurons leads to impaired motor function in a climbing assay. We tested whether MECP2-induced motoneuron dendritic defects affected motor performance of adult flies. First, it is favorable to have an easy to score phenotype to screen potential genetic rescues in future experiments. Second, it is important to test what the functional consequences of the specific dendritic defects reported in this study are. As mentioned above, MN5 is one out of five flight motoneurons innervating the dorsal longitudinal flight muscle (DLM, Fig. 1A). In our experiments, MECP2 was expressed in MN1-5. Therefore, we tested for flight behavioral defects. First, MN5 firing patterns were recorded extracellularly with fine tungsten wires during restrained flight (see methods) [41]. Since *Drosophila* flight is powered by asynchronous flight muscles MN1-5 fire only at about every 10^th^ to 20^th^ wingbeat [49]. No obvious differences were found between MN5 firing patterns during flight in control animals as compared to animals with MECP2 expression in MN1-5 (Fig. 5A). Similarly, wing beat frequencies during flight were not different between both groups (Fig. 5B). Moreover, the likelihood to engage into a flight was not affected by MECP2-induced motoneuron dendritic defects (Figs. 5C, D). Neither the percentage of flies taking off in response to a wind stimulus (Fig. 5C), nor the number of flight bouts that could be elicited in flies were different between controls and MECP2 expressing flies. By contrast, flies with MECP2-caused motoneuron dendritic defects could not maintain flight motor behavior. The mean duration per flight bout (Fig. 5E) was drastically reduced in *MECP2* flies as compared to control flies, on average by a factor of 60. Similarly, total flight duration was significantly reduced in animals with MECP2-caused motoneuron dendritic defects (Fig. 5F), on average by a factor of 30.

## Discussion

### Drosophila as a useful genetic model for studies on MECP2 gain-of-function in neurons

The *Drosophila* genetic model system is experiencing increasing use as a tool to analyze specific genetic and cellular aspects of neurodevelopmental disorders. Short generation times, high fecundity, high throughput screening techniques, facile genetic tools, and relatively low costs have provided valuable mechanistic insights into inherited diseases like Fragile-X, Angelman syndrome, and neurofibromatosis [37]. However, despite considerable conservation in fundamental cell biological pathways the *Drosophila* genome encodes only about 75 percent of human disease associated genes [50], and *mecp2* is not among these genes. Therefore, *Drosophila* can not be used to study the pathophysiology resulting from loss of endogenous *mecp2*. Instead, the *Drosophila* model relies on heterologous expression of human *MECP2* allele and consequential gain of MECP2 function. Although classic Rett is mostly caused by loss-of-function of *MECP2*, this is likely not an artificial approach since in humans and in mouse models increased levels of MECP2 also cause disease [7], [8], [9], [10], [11]. Genetic and behavioral proof of principle for the use of the *Drosophila* model to address MECP2 gain-of-function has recently been provided [40]. In *MECP2* transgenic flies the MECP2 protein associates with chromatin, interacts with homologs of known human MECP2 interactors, modifies the transcription of multiple genes, and is phosphorylated at serine 423, as in mammals. Most importantly, reported consequences are developmental dysfunctions and motor defects, suggesting parallels with RTT phenotypes. However, previous work on MECP2 in the *Drosophila* CNS has not tested for cellular phenotypes resulting from MECP2 over-expression in neurons, although mouse models have demonstrated that disease phenotypes result from *Mecp2* mis-regulation in postmitotic neurons [11]. This study presents the first data on cellular defects as resulting from *MECP2* gain-of-function in developing postmitotic *Drosophila* neurons.

Our data demonstrate that heterologous expression of human MECP2 in *Drosophila* motoneurons does not affect axonal pathfinding, dendritic territory boundaries, or the neurons' electrophysiology, but it causes a significant reduction in new dendritic branch formation during development. Similarly, in the mouse model *Mecp2* mis-regulation results in pyramidal neuron dendritic defects [29], [31]. We provide four lines of evidence that dendritic defects in *Drosophila* motoneurons are caused by specific cellular functions that result from MECP2 gain-of-function, and not from non- specific over-expression or sequestering effects. First, MECP2 protein specifically localizes to the nucleus of *Drosophila* neurons, so that interactions of MECP2 with molecules in the cytoplasm are unlikely. Second, targeted expression of MECP2 in *Drosophila* motoneurons causes significant dendritic branching defects but does not affect firing responses to current injections, voltage activated potassium current, or firing frequencies during motor behavior, indicating normal regulation of electrophysiological properties. Although it has recently been demonstrated that *Drosophila* motoneuron dendritic structure may undergo compensatory changes in response to altered neuronal activity [51], and a link between motoneuron activity and dendritic growth has clearly been established [43], [52], we did not find any evidence for homeostatic changes in motoneuron excitability in response to developmental defects in dendritic structure. Third, MECP2-induced dendritic defects require intact MBD function of the MECP2 protein because dendritic architecture was not affected following expression of *MECP2* alleles with non-functional MBD. This indicates that human MECP2 exerts specific action in *Drosophila* neurons via chromatin remodeling (see below). Fourth, MECP2-induced dendritic phenotypes can be ameliorated by reducing the dose of osa, a member of the SWI/SNF complex. This genetic interaction experiment is consistent with the hypothesis that human MECP2 may exert specific action in *Drosophila* motoneurons via chromatin remodeling. It also indicates that *MECP2* gain-of-function activates specific cell signaling pathways in *Drosophila*, and may not cause unspecific over-expression effects. Therefore, we conclude that *Drosophila* neurons can serve as a valuable model system to identify some cellular mechanisms by which MECP2 gain-of-function affects neuronal development.

### Potential mechanisms underlying MECP2-induced dendritic defects in Drosophila neurons

Our data indicate that dendritic defects as induced by heterologous expression of MECP2 in *Drosophila* motoneurons require an intact MBD domain, because expression of MECP2 with a point mutated or truncated MBD domain has no effects on dendritic structure. However, each *UAS-MECP2* transgene is likely inserted into a unique site in the *Drosophila* genome, and therefore, we can't exclude the possibility that different *UAS-MECP2* transgenes may yield different expression levels or other genetic interactions. For two reasons we judge it unlikely that our finding that dendritic defects as caused by the expression of full length *UAS-MECP2*, but not by the expression of *UAS-MECP2* transgenes with defective MBD domain, were a result of the unique insertion sites of the *UAS-MECP2* constructs into the *Drosophila* genome. First, both UAS-transgenes with defective MBD did not cause dendritic defects. Second, similar dendritic defects were observed following the expression of the full length *MECP2* construct inserted in the second or in the third chromosome.

MBD domains recognize the two key mechanisms of chromatin regulation in eukaryotes, C5 methylations of DNA at cytosines and post-translational histone modifications [47]. Although the existence of DNA methylation has been demonstrated in the fly genome [53], [54], methylation levels are several orders of magnitude lower than in mammals. The fly genome contains only one methylated DNA binding protein (dMBD2/3) and only one DNA methyltransferase (dDNMT2), which shows highest affinity to t-RNA. Consequently, *Drosophila* DNA is only sparsely methylated, so that MECP2 interactions with modified histone tails seem the more parsimonious scenario. This is consistent with our finding that MECP2-dependent dendritic defects are suppressed in an *osa* heterozygous mutant background. Osa is a member of the SWI/SNF complex (human homolog is BAF250), a class of trithorax proteins involved in chromatin remodeling [48] which are highly conserved between flies and humans. This indicates that human MECP2 may exert specific action in *Drosophila* motoneurons via chromatin remodeling. In fact, it has previously been suggested that MECP2 associates with human Brahma, a catalytic component of the SWI/SNF chromatin remodeling complex to regulate gene repression [20], although this finding has been disputed [55]. The *Drosophila* system provides some unique advantages to study possible interactions of MECP2 and members of the SWI/SNF chromatin remodeling complex with genetic tools.

### MECP2-induced motoneuron dendritic defects cause specific motor behavioral deficiencies

Our findings that flies with MECP2 over-expression in motoneurons show normal take-off likelihoods as well as normal motoneuron firing and wing beat frequencies, but can not sustain flight are in accord with specific MECP2 effects on dendrite development in otherwise normal motoneurons. In *Drosophila*, take-off can be mediated by the escape response neural circuitry. This circuitry bypasses flight motoneuron dendrites by synapsing directly on MN5 axon, but it relies on normal synaptic transmission and flight motoneuron physiology [56], [57]. Therefore, initial take-off and initial motoneuron firing are not affected by dendritic defects. In *Drosophila* motoneuron firing frequencies are directly proportional to wing beat frequency [58], and thus, these are also not affected. By contrast, flight can not be sustained because the significantly reduced dendritic surface likely reduces the excitatory synaptic drive to motoneuron dendrites [59] that is necessary to stay in flight. Therefore, flies with MECP2-caused motoneuron dendritic defects show a 30- to 60-fold reduction in flight duration. This behavioral phenotype is obvious, and thus, useful for screening. Although the quantification of flight durations and take-off likelihoods as presented in figure 5 does not allow for rapid genetic screening, high throughput screening can easily be developed based on the observed reduction in flight duration by more than 30-fold. Moreover, high throughput assays which utilize *Drosophila* behavior for rapid screening have been developed by others [60], [61]. Such approaches may help the future identification of candidate MECP2 targets or interactors.

### The use of Drosophila to identify candidate pathways for non-methylated DNA-dependent functions of MECP2 in neurons

Historically MECP2 is viewed as a transcriptional repressor that localizes to chromatin by binding to CpG dinucleotides to regulate gene expression by interactions with histone deacetylases and other cofactors [13], [14], [15], [16]. However, MECP2 also binds to genes that are actively transcribed [17], can associate widely with un-methylated DNA [12], [18], [19], interacts with multiple other proteins [2], and has chromatin compaction and RNA splicing functions [21], [22]. Therefore, multiple MECP2 functions might be mediated by interactions with diverse co-factors and by binding to both methylated and non-methylated DNA. Identification of genetic interactors and modifiers of MECP2 function in neurons will be imperative toward developing future treatment strategies. *MECP2* itself is not a promising treatment target because the X-linked *MECP2* gene is mosaic regulated in the human brain. Furthermore, both loss and gain of function cause disease phenotypes. The sparse methylation landscape in *Drosophila* may offer unique promise of identifying non-methylated DNA-dependent functions of MECP2 in neurons, the cell type that is most relevant to Rett syndrome. Since known binding partners of MECP2 are conserved in flies (e.g. YB-1, mSin3A etc.), it seems plausible that gain-of-function of human *MECP2* may affect neural development via a cellular machinery that is partly conserved between flies and humans.

MECP2-induced dendritic phenotypes in flight motoneurons cause a severe motor behavioral phenotype in that flight bout duration is reduced approximately 30- to 60-fold. Rapid screening assays for *Drosophila* behavioral phenotypes are available [60], [61]. Combined with the fast generation times, high fecundity and facile genetic tools available in *Drosophila* this offers a powerful tool to identify molecules that interact with *MECP2* in neurons. However, potential MECP2 candidate target genes or genetic modifiers of MECP2 function that can readily be identified in the *Drosophila* system will then have to be further evaluated in the existing mouse models of RTT.

## Methods

### Animals

*Drosophila melanogaster* were reared in 68-ml vials on a standard yeast corn meal agar medium at 25°C and 50–60% humidity with a 12-h light/dark regimen. Flies were used for experiments 2 days after eclosion if not stated otherwise. Fly lines that carry different permutations of the human *MECP2* gene as UAS-transgenes were kindly provided by Dr. J Botas (Baylor College of Medicine, Houston, Texas) and were previously published [40]. The first transgene is full-length human *MECP2*, and the other ones show high frequencies of occurrence in patients with RTT. The *R106W* allele is a missense mutation in the methyl-CpG-binding domain (MBD), thus eliminating the protein's ability to bind methylated DNA [46]. In the Δ*166* mutation the MBD and N-terminal portion of the protein are removed. Expression of *UAS-MECP2* transgenes in the motoneuron, MN5, was realized by crossing to recombinant *C380-GAL4, UAS-mCD8-GFP; Cha-GAL80* flies which were obtained from Dr. S Sanyal (Emory University, Atlanta, GA), and have been described previously [43], [52]. *C380* expresses in a subset of motoneurons including MN5, but also in some non-identified sensory neurons and interneurons [62]. Inclusion of the *Cha-GAL80* transgene inhibited expression in cholinergic sensory neurons and interneurons, leaving expression in about thirty neurons per segment in the ventral nerve cord of *Drosophila*, most of which are motoneurons. Given this expression pattern, and the fact that insect motoneurons typically have no output synapses in the central nervous system [63], phenotypes of individual neurons following the expression of *UAS-MECP2* constructs under the control of *C380-GAL4; Cha-GAL80* are likely to result from cell cell autonomous signaling. Therefore, possible indirect effects in motoneurons as resulting from altered neural network properties seem unlikely, although they can't be fully excluded. All morphometric analysis was conducted with female flies. Control data derived from *C380-GAL4, UAS-mCD8-GFP; Cha-GAL80* crossed to *w^1118^* flies and was consistent with quantitative dendritic architecture analysis of MN5 in multiple control strains which has previously been published [44]. Possible interactions between MECP2 and the chromatin remodeling trithorax protein, osa, were investigated by expressing human MECP2 under the control of *C380-GAL4* in a heterozygous mutant background for *osa*. Osa is a member of the SWI/SNF complex (human homolog is BAF250), a class of trithorax proteins involved in chromatin remodeling (Schuettengruber et al., 2007) which are highly conserved between flies and humans. Standard recombination protocols were used to cross *C380-GAL4, UAS-mCD8-GFP;UAS-MECP2; Cha-GAL80* into an osa heterozygous mutant background (*osa^00090^*, loss of function allele, flybase ID: FBal0009367, fly strain 11486 from Bloomington (*ry506 P{PZ}osa00090/TM3, ryRK Sb1 Ser1*).

### Intracellular staining and histology

Thin-walled borosilicate electrodes (resistance of 75–95 MΩ) with filament were used to stain the neurons. Electrode tips were filled with a mixture of 7% Neurobiotin (Linaris GmbH, Wertheim-Bettingen, Germany) and lysine fixable rhodamin-dextran 3000 (Invitrogen, Carlsbad, CA) in 2 M potassium acetate. To prevent dye dilution, an air bubble was left between the tip and the shaft. After intracellular penetration of MN5, the dye was injected iontophoretically by applying constant depolarizing current of 0.5 nA amplitude for 10–12 minutes. Subsequently, the electrode was removed and the ganglia were fixed in 4% paraformaldehyde in phosphate-buffer solution (PBS, 0.1 M, pH 7.4) for 1 h at room temperature and washed in PBS. Preparations were washed 6×30 min in 0.1 M PBS with 0.5% Triton X-100. This was followed by 8 washes, 15 minutes each in PBS. Incubation in Cy3-Streptavidin in PBS (1∶750, Invitrogen, Karlsruhe, Germany) was conducted over night at 4°C. Then, preparations were washed 6×15 min in PBS (0.1 M). Then, the ganglia were dehydrated in an ascending ethanol-series (50, 70, 90, and 100%, 10 min each). Preparations were cleared and mounted in methyl salicylate.

### Electrophysiology

See previous studies for detail [45], [64]. Briefly, wings and legs were cut and the fly was then pinned dorsal side up in a sylgard coated Petri dish and submerged in normal saline (composition in mM: NaCl 128, KCl 2, CaCl_2_ 1.8, MgCl_2_ 4, HEPES 5, sucrose ∼35 depending on the osmolality of the solution). pH was adjusted to 7.25 with 1 M NaOH. Osmolality was adjusted to 290 mOsM/kg with sucrose. The animal was dissected along the dorsal midline, and the large dorsal longitudinal flight muscles were stretched laterally and pinned to expose gut, esophagus, and the ventral nerve cord (VNC) underneath. After removal of the gut and the esophagus, the VNC was exposed. The head was removed to facilitate electrode access to the mesothoracic neuromere. For rapid saline exchange during experiments the volume of the recording chamber was minimized by placing a plexiglas ring (inner diameter 7 mm) around the dissected animal and gluing it to the dish with petrolatum (volume of recording chamber was ∼200 µl). The preparation was then mounted onto an upright fixed stage Zeiss Axioskop 2 FS plus fluorescence microscope (Zeiss, Germany) and viewed with a 40× water immersion objective.

To facilitate access to MN5 with the patch pipette the ganglionic sheath was focally removed with a large patch pipette (0.5 MΩ tip resistance) filled with 2% protease in buffer. This was done under visual control of the flight motoneurons by fluorescent excitation of mCD8-GFP. After protease treatment, the preparation was rinsed with 60 ml normal saline for 10 minutes. Following protease treatment and rinsing, one of the two available MN5s was recorded from with a patch pipette (tip-resistance 5.8–6.5 MΩ) pulled from borosilicate glass (o.d. 1.5 mm, i.d. 1.0 mm without filament from World Precision Instruments) with a vertical pipette puller (Narishige Co., LTD., Japan). For potassium current recordings electrodes were filled with normal internal solution with the following composition (in mM): Kgluconate 140, MgCl_2_ 2, Mg-ATP 2, HEPES 10, EGTA 1.1, glucose to adjust osmolality to 300 mOsM/kg. The pH was adjusted to 7.25 with KOH.

### Immunohistochemistry

Immunohistochemistry was performed as described previously [62]. MN5 intracellular stainings with neurobiotin were visualized by coupling to Cy3-streptavidin (1∶1000) as described previously [43]. Primary antibodies were Mouse anti-MECP2 (1∶1000, AbCam Ab50005), and mouse anti-GFP (1∶400, AbCam Ab1218). The anti-MECP2 antibody was raised against a C-terminal peptide of the MECP2 protein. However, immunostainings with an additional MECP2 antibody that was raised to detect phosphorylated serine 80 in the N-terminal domain of MECP2 (Symansis Cell Signaling Cat # SY-p1205-80) yielded identical results with regard to localization of MECP2 following targeted overexpression (not shown). Secondary antibodies were either Cy2 or Cy5-goat anti-mouse (1∶1000). Incubation, dehydration, clearing and mounting were done as previously described [62].

### Confocal microscopy

Digital images were captured with a Leica TCS SP2 confocal laser scanning microscope (Bensheim, Germany) using a Leica HCX PL APO CS ×40 oil-immersion objective (numerical aperture: 1.2). Intracellular MN5 labeling with neurobiotin and subsequent coupling to Cy-3 streptavidin were scanned with a krypton laser with an excitation wavelength of 568 nm. Emission was detected between 580 and 620 nm. Labels of anti-MECP2 were scanned by using a red HeNe laser at an excitation wavelength of 633 nm, and emission was detected between 640 and 670 nm. Label of anti-GFP was excited with an argon laser at 488 nm and emission was detected between 495 and 530 nm.

### Geometric reconstructions and quantitative morphometry

AMIRA 4.1.1 software (TGS) was used for processing of confocal image stacks. Geometric reconstructions were conducted with custom Amira plug-ins as developed in the Duch lab and described previously [65], [66], [67]. Quantitative morphometric data were imported into Microsoft Excel software and Statistica (StatSoft, Hamburg, Germany) for further analyses. Mann-Whitney-U test was used for comparison of morphometric parameters between two different genotypes and one-way ANOVA was used to determine statistical significance between genotypes for branch order and Sholl analyses. For figure production, images were exported from AMIRA as tiff images and further assembled and labeled in figure panels with CorelDraw13 (Corel Corporation).

The location of MN5 in the CNS is shown in figure 1A, and the overall structure of MN5 is depicted in figure 1B. MN5 is a unipolar cell, and its axon projects into the efferent nerve towards the DLM flight muscle on the contralateral side relative to the cell body. Consequently, axon and cell body are connected by a large primary neurite from which all major dendritic branches arise. To account for this feature in our morphometric analysis, we defined all dendritic branches originating from the primary neurite as first-order branches, virtually eliminating the primary neurite (which is treated as 0-order branch) between cell body and axon and therefore collapsing the reconstruction onto one virtual origin. Every dendritic branch that branches off a first-order branch is defined as a second-order branch, and any branch branching off an *n*-order branch is defined as (*n*+1)-order branch.

### Flight behavioral testing

Behavioral testing was conducted as previously described [68]. Briefly, one day old male flies were immobilized by cold anesthesia for 20 s and glued (clear glass adhesive (Duro; Pacer Technology, Rancho Cucamonga, CA)) with head and thorax to a triangle-shaped copper hook (0.02 mm diameter). Adhesion was achieved by exposure to UV light for 30 s. The animals were then kept individually in small chambers containing a filter paper with 10 µl of a 10% sucrose solution until testing (1–5 h). Then, the fly was attached to the experimental setup via a clamp to accomplish stationary flight. For observation, the fly was illuminated from behind and above (150 W, 15 V; Schott, Elmsford, NY) and fixed in front of a polystyrene panel. Additionally, it was shielded by another polystyrene panel from the experimenter. Tarsal contact with a bead of polystyrene prevented flight initiation before the experiment started. A digital high-speed camera (1000 pictures per second; Motion Scope; Redlake Imaging, Morgan Hill, CA) was positioned behind the test animal. To initiate flight, the fly was gently aspirated. The fly was aspirated as a stimulation to fly each time it stopped flying. When no flight reaction was shown after three consecutive stimulations, the experiment was completed and the total flight time was recorded (extended flight). Every stimulus after the first one, to which the fly showed a response, was recorded. The duration of each flight bout was recorded. Each fly was filmed during the first few seconds of flight, and the recordings were saved on a personal computer for later analysis. The person scoring the flight time was unaware of the treatment group of the animal. All animals were included in the study, including those that did not show any flight behavior.

In some flight experiments, MN5 firing patterns were recorded extracellularly by inserting small tungsten wires (20 µm diameter) into the dorsal most fiber of the DLM flight muscle [41]. Extracellular potentials were amplified 100-fold (AM-Systems 1700), digitized with a 1401 analogue digital converter (Cambridge Electronic Design), and analyzed with Spike2 software (Cambridge Electronic Design).
